# Impact of Immunomodulatory Therapy on COVID-19 Vaccine Response in Patients with Autoimmune Inflammatory Rheumatic Diseases

**DOI:** 10.3390/vaccines12030274

**Published:** 2024-03-06

**Authors:** Ruth Xian Lynn Yap, Yi Wye Lai, Chang Wei, Joel Jia Wei Ng, Dan Xu, Shuo Feng, Rong Mu, Bernard Yu-Hor Thong, Chuanhui Xu

**Affiliations:** 1 Department of Rheumatology, Allergy and Immunology, Tan Tock Seng Hospital, Singapore 308433, Singapore; 2 Department of Rheumatology and Immunology, Peking University Third Hospital, Beijing 100191, China; 3 Yong Loo Lin School of Medicine, National University of Singapore, Singapore 119077, Singapore; 4 Oxford Vaccine Group, Department of Paediatrics, University of Oxford, Oxford OX1 2JD, UK

**Keywords:** vaccination, COVID-19, autoimmune inflammatory rheumatic disease, immunogenicity

## Abstract

Coronavirus disease 2019 (COVID-19) vaccination is essential for patients with autoimmune inflammatory rheumatic diseases (AIIRD) to reduce the risk of morbidity and mortality associated with serious COVID-19 infection. With endemicity, waning of vaccine- and infection-acquired immunity, and development of SARS-CoV-2 variants, the need for additional doses of vaccines against serious illness in high-risk immunocompromised persons remains imperative. This review examines how immunomodulatory therapies affect vaccine-induced immune response in patients with AIIRD. Glucocorticoids, methotrexate, azathioprine, calcineurin inhibitors, mycophenolate mofetil, tumor necrosis factor inhibitors, and abatacept have been shown to variably attenuate both humoral and cellular immune responses to vaccination. Janus kinase inhibitors reduce humoral immune response. In contrast, sulfasalazine, leflunomide, belimumab, interleukin (IL)-17, IL-12/23, IL-6, and IL-1 inhibitors appear favorable, with mild or no impact on vaccine response. Although rituximab is known to profoundly diminish humoral immune response, cellular immunity is relatively preserved. Administering a third and subsequent vaccine dose or temporally coordinating the dosing of immunomodulatory drugs may improve vaccine effectiveness. Further research is needed to personalise vaccination strategies for AIIRD patients, considering their specific immunomodulatory treatments.

## 1. Introduction

Patients with autoimmune inflammatory rheumatic diseases (AIIRD) are at increased risk of infections and severe clinical outcomes, primarily due to their immunocompromised status and the presence of comorbidities [1,2,3]. The coronavirus disease 2019 (COVID-19) pandemic has exacerbated these risks, posing an unprecedented health threat to this patient population globally [4,5]. In this context, vaccination emerges as a critical strategy, providing protection by inducing the adaptive immune response against vaccine-preventable serious illness. Therefore, it is imperative to ensure that patients with AIIRD receive COVID-19 vaccinations to mitigate infection-related morbidity and mortality.

Immunogenicity, encompassing both humoral and cellular immune responses, is often used as a surrogate to evaluate vaccine efficacy [6]. In AIIRD patients, the use of immunomodulatory drugs not only contributes to their immunocompromised status but also potentially diminishes the immunogenicity and overall efficacy of vaccinations. Furthermore, this patient group frequently exhibits vaccine hesitancy due to the lack of confidence in vaccine efficacy and the concerns over vaccine safety, in particular the triggering flares of AIIRD.

It is important to understand the impact of immunomodulatory drugs on the immunogenicity of vaccination in patients with AIIRD. However, the evidence in this area remains limited and complicated by the heterogeneity of immunomodulatory drugs in terms of mode of action, dosage and different permutations/combinations of such therapies, different patient phenotypes and endotypes, and different types of vaccine platforms available during the different phases of the pandemic (messenger ribonucleic acid [mRNA] vaccines and non-mRNA vaccines).

This review aims to summarise the existing evidence to inform rheumatologists and other healthcare providers about the impact of immunomodulatory drugs on COVID-19 vaccine immunogenicity in adult AIIRD patients. It seeks to provide insights into optimising the strategies of COVID-19 vaccination for this vulnerable group. Most of the studies (unless otherwise stated) described the experience with mRNA vaccines (Pfizer—BioNTech and Moderna) with a few studies which included the adenovirus vector vaccine (Oxford—AstraZeneca), and other inactivated virus vaccines.

## 2. Glucocorticoids

Glucocorticoids are known for their potent anti-inflammatory effects via both genomic and nongenomic pathways [7]. Glucocorticoids suppress the innate and adaptive immune systems by altering the transcription of various glucocorticoid-responsive genes, leading to downstream effects [7]. Given their extensive immunomodulatory effects, it is not unexpected that glucocorticoids have been demonstrated to attenuate the humoral and cellular immune responses elicited by the COVID-19 vaccine.

Studies have shown both qualitative and quantitative decreases in the humoral response to the COVID-19 vaccine with glucocorticoid use. A multicentre observational study of 686 patients with AIIRD by Furer et al. found that the overall seropositivity rate was 86% (*n* = 590) in patients with AIIRD and 66% in those using glucocorticoids (*n* = 130, *p* < 0.0001), although a majority of the patients (117 out of 130) were concurrently on another immunosuppressant [8]. Among the 13 patients with glucocorticoid monotherapy, the seropositivity rate was 77% [8]. Similar findings were reported by other studies [9,10,11,12], including one where inactivated vaccines were also used [9]. In a study of 597 patients with AIIRD who received homologous inactivated vaccine, Aikawa et al. demonstrated that even low doses of prednisolone (5 mg per day) were associated with reduced anti-SARS-CoV-2 S1/S2 IgG positivity (odds ratio (OR) = 0.46, 95% confidence interval (CI): 0.27 to 0.77, *p* = 0.003) and neutralising antibody positivity (OR = 0.63, 95% CI: 0.44 to 0.90, *p* = 0.011) [13]. Several observational studies support the association between a daily prednisolone equivalent dose above 7.5 mg and decreased neutralising antibody responses following both inactivated and mRNA vaccines [14,15].

Research has demonstrated the impact of glucocorticoid use on cellular immunity in AIIRD patients. Renaudineau et al. observed reduced T cell responses (evaluated by interferon-gamma release assay (IGRA) against spike proteins, IGRA-S) in patients receiving glucocorticoids. In their single-centre retrospective study of 87 patients with AIIRD who received mRNA and viral vector (Oxford—AstraZeneca) vaccines, 23 patients (26.4%) showed a negative IGRA response. IGRA-S-negative patients were predominantly (18/23) treated with glucocorticoids [16]. Interestingly, the glucocorticoid capacity to inhibit T cell-mediated immune response was independent of dose per day, cumulative dose, time from glucocorticoid initiation, and the synthetic glucocorticoid that was used [16]. Furthermore, in a study by Saleem et al. of 100 patients with rheumatoid arthritis who received mRNA and viral vector vaccines, there was a trend towards a better development of T cell responses in patients who had not recently received glucocorticoids (OR = 0.28, 95% CI: 0.07–1.18, *p* = 0.083) [17]. Similarly, Yang et al. found that glucocorticoid use was associated with a lower rate of IGRA response following mRNA and non-mRNA vaccines [18].

## 3. Conventional Synthetic Disease-Modifying Antirheumatic Drugs (DMARDs) and Immunosuppressive Therapies

### 3.1. Methotrexate

Methotrexate (MTX) is widely used in AIIRD. It inhibits dihydrofolate reductase, thereby preventing the reduction of dihydrobiopterin (BH2) to tetrahydrobiopterin (BH4). This leads to nitric oxide synthase uncoupling and increased sensitivity of T cells to apoptosis [19]. It also increases adenosine release which then activates adenosine receptor A2a. These mechanisms inhibit the activation of nuclear factor-κB (NF-κB) and serve to diminish immune responses [19].

Various studies have reported reduced humoral response to mRNA and inactivated virus vaccines in patients with AIIRD receiving MTX [8,20,21,22,23,24]. Furer et al. found that the seropositivity rate measured 2–6 weeks after the second vaccine dose (Pfizer—BioNTech BNT162b2 or Moderna mRNA-1273) was 92% in patients on MTX monotherapy compared with 100% in controls (*p* = 0.02) [8]. Similarly, Moyon et al. found that MTX use in SLE patients was associated with lower anti-spike antibody production measured 14 days after the second dose (β = −122, 95% CI: −184 to −61, *p* < 0.001) and reduced neutralising antibody (β = −1.1, 95% CI: −1.9 to −0.34, *p* < 0.001) [20]. MTX in combination with other immunosuppressants also significantly reduced seroconversion rates compared to that in controls (84% vs. 100%, *p* < 0.0001), although at a smaller magnitude than with anti-CD20, mycophenolate mofetil (MMF), and abatacept treatments [8]. MTX when used in combination with tumor necrosis factor inhibitors (TNFi) reduced the rate of seropositivity to 93% (*p* = 0.04) [8]. A cohort study suggested that although the early immunosuppressive of MTX was attenuated in patients with concomitant TNFi use, at 6 months this combination had the lowest adequate humoral response rate of any medication even when compared to all MTX use (alone or in combination with other medications) [25].

There were a few studies on the impact of MTX on the cellular immune response in AIIRD patients receiving the COVID-19 vaccine. In a study by Moyon et al., the T cell responses measured by IGRA following SARS-CoV2 spike protein stimulation was 57% in patients with detectable neutralising activity, and only 10% in those without detectable neutralising activity [20]. Another observational study demonstrated reduced CD4 T cell response (interleukin(IL)-2 and interferon-gamma (IFN-γ) production) and CD8 T cell response (S-induced Granzyme A/B detection) in MTX compared to TNFi use, leflunomide use, and healthy controls (CD4 T cell response: 80% vs. 100%, 100% and 100%, respectively; CD8 T cell response: 76% vs. 100%, 100% and 92%, respectively) [26]. Compared with healthy adults and AIIRD patients not on MTX, the production of activated CD8 T cells (expressing Ki67 and CD38) and the granzyme B-producing subset of these activated CD8 T cells were not induced in AIIRD patients on MTX [23].

Various treatment modification strategies have been proposed to improve the immunogenicity of the SARS-CoV-2 vaccine in AIIRD patients on MTX. While the American College of Rheumatology (ACR) Guidance recommended withholding MTX for 1–2 weeks after each of the two mRNA vaccine doses, Moutsopoulos et al. recommended withholding anti-metabolites 10 days before and 10 days after each vaccination dose [27,28]. Tzioufas et al. found that AIIRD responders either on MTX monotherapy or combined MTX-based regimes underwent more frequently extended treatment modifications as Moutsopoulos recommended than non-responders (21.49 vs. 4.28%, OR = 6.115, 95% CI: 2.033–18.91, *p* = 0.0001) [9]. These patients developed antibody titres that were comparable with patients without immunomodulatory therapies and significantly higher than patients on immunomodulatory therapies that were not suspended [9]. The VROOM study was a randomised open-label superiority trial which showed that a 2-week interruption of MTX in AIIRD patients improved S1-receptor binding domain (RBD) antibody response. However, the authors cautioned about the high risk of bias due to the exclusion of participants after randomisation for previous SARS-CoV-2 infections and disease flare-ups, a dropout rate of 33%, and different dropout rates in the suspended MTX group compared to the continued treatment group [29]. A single-centre open-labeled, assessor-masked, randomised controlled trial in India demonstrated that the final antibody titres were higher among patients with either psoriatic arthritis or rheumatoid arthritis who withhold MTX for 2 weeks after both doses or only after the second dose of ChAdOx1 nCov-19 (Oxford—AstraZeneca) vaccine compared to those who did not withhold MTX. Of interest, the humoral response was similar between patients withholding MTX for 2 weeks after both doses or only after the second dose of ChAdOx1 nCov-19 (Oxford—AstraZeneca) vaccine, and fewer flare-ups of arthritis in the latter group [30].

### 3.2. Sulfasalazine

Sulfasalazine, metabolised in vivo into active compounds, exerts anti-inflammatory and anti-bacterial effects, although the precise mechanism of action remains uncertain. In patients with axial spondyloarthritis and psoriatic arthritis, those on sulfasalazine monotherapy displayed 100% seropositivity rates and 83.3% neutralising antibody positivity after three vaccinations, showing no significant differences with the control group (*p* > 0.999) [31]. While TNFi monotherapy reduced seropositivity (76.3% vs. 96%; *p* < 0.001) and neutralising antibody positivity (47.4% vs. 83%, *p* < 0.001) compared to the control group, its combination with sulfasalazine did not significantly impair the humoral immune response [31]. This suggests that the immunomodulatory effect of sulfasalazine may counterbalance the dampened vaccine humoral response with TNFi [31].

### 3.3. Leflunomide

Leflunomide is an inhibitor of pyrimidine synthesis. It acts by blocking dihydroorotate dehydrogenase. Evidence on the impact of leflunomide on the immunogenicity of COVID-19 vaccination is scarce. In a study that involved 10 patients with AIIRD on leflunomide monotherapy, the seropositivity rate was 100% after two doses of mRNA COVID-19 vaccination, with a comparable titre of anti-spike antibodies as in healthy controls [26]. However, Aikawa et al. found that negative neutralising antibodies were associated with leflunomide treatment (*p* = 0.016) [15]. Leflunomide treatment was independently associated with negative neutralising antibodies after four vaccinations (OR = 0.32, *p* = 0.036) [15].

### 3.4. Azathioprine

Azathioprine, a purine analogue, inhibits lymphocyte proliferation by blocking purine synthesis, thereby dampening inflammation [32]. Santos et al. observed a seroconversion rate of 57% in AIIRD patients on azathioprine following two vaccine doses (mRNA SARS-CoV-2 vaccine). Azathioprine was shown to impair humoral responses with reduced antibody titres compared to healthy controls (178.5 ± 142 vs. 526.3 ± 2078 BAU/mL, *p* = 0.01) [26]. Additionally, only 42% of these patients exhibited a complete cellular response, significantly lower than the 96% average in healthy controls, although this finding is based on a small sample size of seven [26]. In another study, Carruthers et al. reported a seroconversion rate of 87.5% in patients taking azathioprine for ANCA-associated vasculitis [33].

### 3.5. Cyclosporin/Tacrolimus

Calcineurin inhibitors, such as cyclosporin and tacrolimus, are known to inhibit T cell proliferation [32]. A prospective observational study by Zecca et al. identified calcineurin inhibitors as an independent predictor of impaired humoral responses and vaccination failure in patients, with an odds ratio of 17.5 (95% CI: 3.1–99.0, *p* = 0.0012) for vaccination failure, although the risks may be confounded by concomitant MMF treatment [34]. Prendecki et al. reported a 67.7% seroconversion rate among patients with autoimmune rheumatic and glomerular diseases treated with tacrolimus [35]. While the proportion of cellular responses in tacrolimus patients did not significantly differ from non-tacrolimus patients, the magnitude of this response was significantly lower in the former group [35].

### 3.6. Mycophenolate Mofetil

MMF, a prodrug of mycophenolic acid, blocks de novo purine synthesis, thereby reducing cell proliferation [32]. MMF treatment has been shown to significantly reduce seroconversion rates (69% in MMF group vs. 100% in healthy controls, *p* < 0.001) and antibody titres in response to the Moderna COVID-19 (mRNA1273) vaccine, with a notable dose-dependent effect. This effect was most pronounced in patients receiving over 1 g daily, leading to both reduced neutralising activity and impaired cytotoxic cellular response [36]. A similar dose-dependent impact on humoral immune response was observed in another study [37]. Sieiro et al. showed that 70% of patients on MMF showed a complete cellular response after a two-dose mRNA COVID-19 vaccine [26]. Additionally, Wieske et al. found that a third vaccine dose could enhance seroconversion rates in MMF-treated patients, increasing from 52.6% after the second dose to 89.5% after the third [38].

### 3.7. Cyclophosphamide

Cyclophosphamide is a cytotoxic drug that alkylates deoxyribonucleic acid (DNA), inducing DNA cross-links and thereby leading to cell death, primarily in proliferating cells. There is no data on the effect of concurrent cyclophosphamide treatment on immune responses after COVID-19 vaccination. However, Carruthers et al. found that past cyclophosphamide treatment, where the last treatment was given more than 12 months prior to vaccination, did not affect seroconversion after vaccinations. The seroconversion rate of the 29 patients with historic treatment was 82.8%, whereas the seroconversion rate in those unexposed to treatment (*n* = 17) was 82.6% (*p* > 0.99) [33].

## 4. Biologic Disease Modifying Anti-Rheumatic Drugs (DMARDs)

### 4.1. Tumor Necrosis Factor Inhibitors (TNFi)

TNFi are widely used to treat AIIRD such as rheumatoid arthritis, ankylosing spondylosis, and psoriatic arthritis. While some studies have not shown an impaired humoral response with TNFi use [9,39,40,41], other studies showed a higher risk of non-seroconversion and reduced antibody titres [25,31,38,42,43]. The seroconversion was 90.7% in patients with AIIRD receiving TNFi monotherapy in an observational study, 83.3% for tocilizumab monotherapy, and 100% for IL-17 inhibitors monotherapy, respectively [42]. In a prospective cohort study of spondyloarthropathy with age and sex-matched control group, TNFi monotherapy was associated with the absence of seroconversion (OR = 0.21, 95% CI: 0.05–0.90) [31]. Additionally, anti-RBD immunoglobulin G (IgG) titres were significantly lower in TNFi recipients compared with healthy controls (median 726 AU/mL and 3355 AU/mL, respectively, *p* < 0.001) [43]. Haberman et al. found a reduced likelihood of an adequate humoral response at 6 months following vaccination in patients on TNFi (OR = 0.48, 95% CI: 0.26–0.90, *p* = 0.022), although no differences were found at 4 weeks and 3 months [25].

TNFi appears to impair cellular immunity [24]. The T cell response was 79% in TNFi-treated patients with psoriasis compared to 100% in healthy controls after two doses of the COVID-19 vaccine [24]. A study involving inflammatory bowel disease patients treated with infliximab (*n* = 2279) showed moderate effects on humoral immunity, including antibody concentrations and neutralising capacity compared with those treated with vedolizumab (*n* = 1031) [44]. The proportion of patients who did not develop detectable T cell responses was comparable between both groups (infliximab 19.6% vs. vedolizumab 19.2%) [44]. A retrospective study of 11,468 vaccinated AIIRD patients found that TNFi use was associated with an increased risk of breakthrough infections with a hazard ratio of 1.70 [45].

### 4.2. Rituximab

Rituximab is a chimeric monoclonal antibody targeting CD20-positive B cells. It has been reported to significantly impair the humoral response to COVID-19 vaccination. A multicentre cohort study reported a seropositivity rate of 39%, the lowest compared with other immunosuppressants, further decreasing to 36% when combined with methotrexate [8]. The seropositivity rate improved over time since the completion of rituximab, increasing from below 20% within 6 months after rituximab treatment to about 50% for vaccinations administered 1 year post-treatment [8]. Rituximab was associated with poor vaccine response to COVID-19 mRNA vaccination (OR = 6.80, 95% CI: 1.93–24.3, *p* = 0.005) [9]. An observational cohort study by Sierio et al. reported a 31% seroconversion rate among AIIRD patients on rituximab, with notably lower anti-spike IgG titres [26]. A meta-analysis including 4423 participants in 11 countries showed a negative impact of rituximab on the seroconversion rate of COVID-19 vaccination in patients with inflammatory rheumatic diseases. The serological response rate was only 36.3% (95% CI: 28.6–44.4%), which is the lowest among patients treated with immunomodulatory agents [41]. Predictive factors for a better humoral response included the time since the last anti-CD20 therapy (>7.6 months), peripheral CD19^+^ cell count (>27 cells per μL), and CD4^+^ lymphocyte count (>653 cells per μL) [46].

While rituximab’s impact on cellular immunogenicity was not significantly different from systemic rheumatic disease patients not on immunosuppressants, it was reduced compared to healthy controls [26]. In rituximab-treated serological non-responders, CD4 T cell and CD8 T cell responses were often observed [26]. The findings of reduced cellular immunogenicity compared with healthy controls were also demonstrated in a study by Moor et al., where SARS-CoV-2-specific IFNγ release was detected in 20% of anti-CD20 treated patients versus 75% of healthy controls (*p* < 0.001) [46].

Considering the poor antibody response in rituximab-treated patients, additional vaccination strategies are being explored to enhance COVID-19 protection. Rose et al. studied the effect of a supplemental COVID-19 vaccine in a cohort of rituximab-treated patients who underwent two doses of vaccination [47]. They found that 53.1% of these patients developed detectable SARS-CoV-2 spike protein IgG antibodies post supplemental dose, compared to only 24.1% after the initial primary series [47]. The ACR currently recommends completing the vaccination series approximately 4 weeks prior to the next rituximab dose based on the data on influenza vaccination responses after rituximab [27]. Additionally, a retrospective cohort study comparing rituximab-exposed and rituximab-naïve patients with immune-mediated dermatologic disease suggested that a nine-month interval between rituximab administration and vaccination optimises immunogenicity while avoiding unnecessary delay in treatment [48].

### 4.3. Belimumab

Belimumab is a monoclonal antibody targeting B lymphocyte stimulator (BlyS) to inhibit B cell survival. Most studies have shown good seroconversion rates for patients on belimumab after the vaccine [41,49,50]. Fabris et al. observed a 94.1% seroconversion rate after two vaccine doses, albeit with lower antibody titres compared to healthy controls [49]. Boedecker-Lips et al. reported a 90% seroconversion rate after three vaccine doses [50]. A meta-analysis by Auroux et al., covering 113 patients on belimumab, showed a seroconversion rate of 84.3% (95% CI: 65.5–96.6) [41]. However, Aikawa et al. found that belimumab treatment was negatively associated with neutralising antibody positivity (OR = 0.29, 95% CI: 0.13 to 0.67, *p* = 0.004) [13]. Regarding cellular immunity, Fabris et al. reported that the cellular response in belimumab-treated patients was comparable to that of healthy controls, indicating that T cell response remains relatively unaffected by belimumab [49].

### 4.4. Interleukin-17 Inhibitors

IL-17 inhibitors, which are monoclonal antibodies targeting IL-17 activity, are used to treat psoriasis, psoriatic arthritis, and ankylosing spondylitis. Current studies indicate that IL-17 inhibitors do not impair the humoral response, either as monotherapy or in combination [24,31,51,52]. Andreica et al. showed that patients on IL-17 inhibitors had significantly higher neutralising antibodies compared to those on other DMARDs [53]. Smetanova et al. observed 100% seroconversion in spondyloarthritis patients treated with secukinumab monotherapy, with anti-SARS-CoV-2-specific IgA and IgG antibodies comparable to healthy individuals [51]. A meta-analysis of observational studies reported a 97.3% (95% CI: 93.9–99.3) seroconversion rate in AIIRD patients on IL-17 inhibitors [41].

Regarding cellular immunity, Andreica et al. found no impairment in patients on IL-17 inhibitors [53]. Smetanova et al. also observed similar cellular responses between secukinumab-treated patients and healthy controls [51]. An interim study by Mahil et al. found that cellular immunity in psoriasis patients on IL-17 inhibitors was comparable to healthy control after the first dose of the COVID-19 vaccine [52]. However, a subsequent study by the same group noted that 38% of patients on IL-17 inhibitors did not exhibit cellular responses after a second vaccine dose, although the magnitude of the responses in the remaining 62% of responders was not significantly different from healthy controls [24].

### 4.5. Interleukin-12/23 Inhibitors

IL-12/23 inhibitors, which are monoclonal antibodies targeting both IL-12 and IL-23 activity, are used to treat psoriasis, psoriatic arthritis, and inflammatory bowel disease. A study on spondyloarthritis patients treated with ustekinumab reported no significant impact on seroconversion and level of neutralising antibodies, although the sample size was small [31]. An interim study by Mahil et al. showed an initial seroconversion rate of 83% (95% CI: 61–95) in psoriasis patients on IL-23 inhibitor monotherapy post the first vaccine dose, lower than the 100% rate in healthy controls [52]. However, this rate increased to 100% after the second vaccine dose, suggesting that IL-23 inhibitor monotherapy does not impair humoral immunity after two doses [24].

With regard to cellular immunity, about 22% of psoriasis patients on IL-23 inhibitors did not display cellular responses post the second vaccine dose, similar to observations with IL-17 inhibitors, compared to 100% in healthy controls [24]. Nevertheless, the magnitude of the cellular responses in the remaining 78% of patients on IL-23 inhibitors was comparable to that of healthy controls [24].

### 4.6. Interleukin-6 Receptor Inhibitors

IL-6 receptor inhibitors are monoclonal antibodies that block IL-6 activity, including tocilizumab and sarilumab. Studies by Tzioufas et al. and Monti et al. reported that tocilizumab did not adversely affect the humoral response following two COVID-19 vaccinations in AIIRD patients [9,54]. Le Moine et al. observed a seroconversion rate of 85.7% in rheumatoid arthritis patients after two vaccinations [40]. Auroux et al., however, reported a high seroconversion rate of 96.3% in their AIIRD cohort, and a subsequent meta-analysis indicated a seroconversion of 93.9% (95% CI: 81.5–99.7) for IL-6 inhibitors, suggesting that these inhibitors do not significantly affect the humoral response to the vaccine [41]. Additionally, tocilizumab was not found to affect cellular responses in patients with giant cell arteritis [54].

### 4.7. Interleukin-1 Inhibitors

A meta-analysis reported a high seroconversion rate of 97% (95% CI: 76.5–100) in patients with AIIRD who received two COVID-19 mRNA or AstraZeneca vaccinations while on IL-1 receptor antagonists. This analysis, however, was based on the serological responses of only 15 patients across three studies [41]. Data on the cellular immune response to COVID-19 vaccine in patients treated with IL-1 inhibitors are currently lacking.

### 4.8. Abatacept

Abatacept, a cytotoxic T-lymphocyte associated protein 4 (CTLA-4) IgG fusion protein with high affinity for CD28, limits T cell costimulation by competing with CD28 for binding to CD80 and CD86 [55]. Research indicated that abatacept therapy significantly diminished the immune response to COVID-19 vaccinations [56]. This observation was corroborated by Saleem et al., who reported that none of the eleven patients treated with abatacept therapy had seroconversion, compared to 100% for healthy controls [17]. Additionally, patients on abatacept therapy showed decreased titres of IgG anti-RBD antibodies (*p* = 0.02) [37]. Furer et al. found that the serum IgG neutralising antibody levels were lower, which were measured 2–6 weeks post the second vaccine dose [8]. A third vaccine dose appeared to enhance the humoral response in these patients [57].

Abatacept is also associated with reduced cellular immunogenicity, where only 10% of participants demonstrated T cell responses [26]. Moreover, abatacept treatment is linked to a higher risk of infection compared to antimalarial monotherapy, with an adjusted hazard ratio of 3.52 (95% CI: 1.90, 6.51) [58].

## 5. Targeted Synthetic DMARDs/JAK Inhibitors

JAK inhibitors, which block the JAK-signal transducer and activator of the transcription (STAT) signalling pathway, have been shown to affect vaccination responses. Schäfer et al. reported a markedly lower antibody response to COVID-19 vaccination in patients treated with JAK inhibitors, with only 54.9% maximum responders, compared to 77.4% in controls not receiving DMARD therapy. Notably, the combined treatment of JAK inhibitors and MTX resulted in further diminished immune responses than monotherapy with JAK inhibitors [59]. In contrast, Seror et al. observed a high overall COVID-19 vaccine response rate in the treated JAK inhibitors, with an 88% (100/113) seropositivity rate [60]. Moreover, the humoral response demonstrated a resurgence following a third vaccine dose [57]. Currently, there is no information available on the cellular immune response to vaccination in patients with JAK inhibitors. Additionally, Patel et al. indicated that JAK inhibitors were associated with increased infection risks compared to antimalarial monotherapy [45].

## 6. Conclusions

This review highlights the impact of immunomodulatory drugs on the immunogenicity of COVID-19 vaccination in AIIRD patients (Table S1). Overall, the impact of immunomodulatory drugs on COVID-19 vaccine immunogenicity and the risk of infection in AIIRD patients varies widely depending on the specific medication. Various medications, including glucocorticoids, MTX, azathioprine, calcineurin inhibitors, MMF, TNFi, and abatacept have been found to reduce both humoral and cellular immune response following vaccination. JAK inhibitors lower humoral immune response. On the other hand, sulfasalazine, leflunomide, belimumab, and inhibitors of IL-17, IL-12/23, IL-6, and IL-1 seem to have a mild or no effect on vaccine immunogenicity. While rituximab significantly decreases the humoral immune response, the cell-mediated immunity is relatively preserved. TNFi, abatacept, and JAK Inhibitors treatment were associated with an increased risk of COVID-19 infection.

## 7. Discussion and Future Perspectives

A few strategies may be considered to enhance vaccine responses and optimise vaccination efficacy among this vulnerable population. Firstly, an enhanced primary series of COVID-19 vaccination with an additional dose has been recommended as the standard of care in several jurisdictions [61]. Secondly, the interruption of immunomodulatory drugs where possible could improve the immunogenicity of vaccination, in accordance with ACR recommendations [27] or national guidelines. However, this strategy requires careful consideration of the individual’s disease activity and risk of flare. The development of personalised vaccination strategies tailored to the specific treatment of AIIRD patients warrants further investigation.

The majority of current evidence is based predominantly on peak immune responses that occur a few weeks post-vaccination. The effects of immune waning and the utility of booster campaigns in the AIIRD population, however, require more long-term data. At present, this is still limited. Given the ongoing circulation of SARS-CoV-2, this represents a crucial gap in our understanding of the durability of vaccine-induced immunity in AIIRD patients, especially those on medications like MTX, leflunomide, or rituximab, which are shown to dampen initial immune responses. Additionally, the long-term monitoring of vaccine-optimising strategies is imperative, such as withholding MTX around vaccination as suggested by the ACR and Moutsopoulos et al. [28]. Understanding these dynamics is critical for effectively tailoring COVID-19 vaccination strategies to meet the unique needs of AIIRD patients.

Our review is subject to a few limitations. The measurements of immune responses, including humoral and cellular responses, were not often standardised and therefore might introduce inconsistencies in definitions and results. Additionally, the number of studies included in this review may be affected by the prescribing pattern for immunomodulatory therapies, with newer or less commonly prescribed being less represented. Moreover, limiting our search to English-language publications may have resulted in the omission of data from studies published in other languages.

The emerging evidence from SARS-CoV-2 vaccination during the COVID-19 pandemic provides an opportunity to understand the impact of immunomodulatory drugs on the efficacy of vaccination in adult patients with AIIRD. This may guide rheumatologists and other healthcare providers to optimise other common vaccination protocols, i.e., influenza and pneumococcal vaccines, in AIIRD patients. As such, this evidence provides useful guidance for rheumatologists and other healthcare professionals in making informed decisions about COVID-19 vaccination strategies for individuals with AIIRD, potentially contributing to improved patient outcomes and more effective vaccination approaches.

## Supplementary Materials

The following supporting information can be downloaded at: https://www.mdpi.com/article/10.3390/vaccines12030274/s1, Table S1: Summary of the impact of immunomodulatory therapy on COVID-19 vaccine response in patients with AIIRD.
