# Supplementary material for: Impact of Immunomodulatory Therapy on COVID-19 Vaccine Response in Patients with Autoimmune Inflammatory Rheumatic Diseases
Source: Vaccines (Basel). 2024 Mar 6;12(3):274. doi: 10.3390/vaccines12030274 (PMC10974321; doi:10.3390/vaccines12030274)
Supplement: Supplementary file 1 [file vaccines-12-00274-s001.zip › vaccines-2837422-supplementary.pdf]

Table S1. Summary of the impact of immunomodulatory therapy on COVID-19 vaccine response in patients with AIIRD.

Drugs	Mechanism of action	Humoral immune response	Cellular immune response	Strategies to hold off drugs
Glucocorticoids	Broad anti-inflammatory effect via both genomic and nongenomic pathways	↓	↓	
MTX	Reduce lymphocyte proliferation via inhibiting dihydrofolate reductase, and inhibit NF-κB pathway by increasing adenosine release	↓	↓	Withholding MTX for 1-2 week after each of the 2 mRNA vaccine doses[1-3]
Sulfasalazine	Unclear	-	Uncertain	
Leflunomide	Reduce expansion of activated lymphocytes by inhibiting pyrimidine synthesis	- or ↓	Uncertain	
Azathioprine	Inhibit lymphocyte proliferation by blocking purine synthesis	↓	↓	
Cyclosporin/ Tacrolimus	Reduce T cell proliferation as calcineurin inhibitor	↓	↓	
Mycophenolate mofetil	Reduce cell proliferation by blocking de novo purine synthesis	↓	↓	

Cyclophosphamide	Promote cell death of proliferating cells by inducing DNA cross-link	Uncertain	Uncertain	
TNF inhibitors	Inhibit tumor necrosis factor	↓	↓	
Rituximab	Deplete CD20+ B cells	↓↓	- or ↓	Nine month interval between rituximab administration and vaccination[4]; 4 weeks prior to next rituximab dose[2]
Belimumab	Reduce survival of autoreactive B cells by inhibiting B lymphocyte stimulator (BlyS)	- or ↓	-	
IL-17 inhibitors	Inhibit IL-17 activity	-	-	
IL-12/23 inhibitors	Inhibit both IL-12 and IL-23 activity	-	-	
IL-6 receptor inhibitors	Inhibit IL-6 pathway	-	-	
IL-1 inhibitors	Inhibit IL-1 pathway	-	Uncertain	
Abatacept	Inhibit T cell activation by binding to CD80/CD86 on antigen-presenting cells	↓	↓	
JAK inhibitors	blocking JAK-STAT signalling pathway	↓	Uncertain	

Abbreviations: COVID-19: Coronavirus disease 2019. AIIRD: autoimmune inflammatory rheumatic diseases. NF-κB: nuclear factor-κB. MTX: methotrexate. mRNA: messenger Ribonucleic Acid. TNF inhibitors: Tumor necrosis factor inhibitors. IL: interleukin. JAK: Janus Kinase. JAK-STAT: Janus Kinase-Signal Transducer and Activator of Transcription.

1. **Abhishek A, Boyton RJ, Peckham N, McKnight A, Coates LC, Bluett J, Barber V, Cureton L, Francis A, Appelbe D *et al*: Effect of a 2-week interruption in methotrexate treatment versus continued treatment on COVID-19 booster vaccine immunity in adults with inflammatory conditions (VROOM study): a randomised, open label, superiority trial. *Lancet Respir Med* 2022, 10(9):840-850.**
2. **Curtis JR, Johnson SR, Anthony DD, Arasaratnam RJ, Baden LR, Bass AR, Calabrese C, Gravallese EM, Harpaz R, Kroger A *et al*: American College of Rheumatology Guidance for COVID-19 Vaccination in Patients With Rheumatic and Musculoskeletal Diseases: Version 5. *Arthritis & Rheumatology* 2023, 75(1):E1-E16.**
3. **Skaria TG, Sreeprakash A, Umesh R, Joseph S, Mohan M, Ahmed S, Mehta P, Oommen SE, Benny J, Paulose A *et al*: Withholding methotrexate after vaccination with ChAdOx1 nCov19 in patients with rheumatoid or psoriatic arthritis in India (MIVAC I and II): results of two, parallel, assessor-masked, randomised controlled trials. *The Lancet Rheumatology* 2022, 4(11):e755-e764.**
4. **Seree-Aphinan C, Ratanapokasatit Y, Suchonwanit P, Rattanakaemakorn P, P OC, Pisitkun P, Suangtamai T, Setthaudom C, Chirasuthat S, Chanprapaph K: Optimal time for COVID-19 vaccination in rituximab-treated dermatologic patients. *Front Immunol* 2023, 14:1138765.**
